# *In Vitro* Activity of the Antifungal Azoles Itraconazole and Posaconazole against *Leishmania amazonensis*

**DOI:** 10.1371/journal.pone.0083247

**Published:** 2013-12-23

**Authors:** Sara Teixeira de Macedo-Silva, Julio A. Urbina, Wanderley de Souza, Juliany Cola Fernandes Rodrigues

**Affiliations:** 1 Laboratório de Ultraestrutura Celular Hertha Meyer, Instituto de Biofísica Carlos Chagas Filho, Universidade Federal do Rio de Janeiro, Rio de Janeiro, Brazil; 2 Instituto Nacional de Ciência e Tecnologia de Biologia Estrutural e Bioimagem, Rio de Janeiro, Brazil; 3 Instituto Venezolano de Investigaciones Científicas, Centro de Bioquímica y Biofísica, Caracas, Venezuela; 4 Instituto Nacional de Metrologia, Qualidade e Tecnologia, Inmetro, Rio de Janeiro, Brazil; 5 Núcleo Multidisciplinar de Pesquisa em Biologia (NUMPEX-BIO), Polo Avançado de Xerém, Universidade Federal do Rio de Janeiro, Duque de Caxias, Brazil; Royal Tropical Institute, The Netherlands

## Abstract

Leishmaniasis, caused by protozoan parasites of the *Leishmania* genus, is one of the most prevalent neglected tropical diseases. It is endemic in 98 countries, causing considerable morbidity and mortality. Pentavalent antimonials are the first line of treatment for leishmaniasis except in India. In resistant cases, miltefosine, amphotericin B and pentamidine are used. These treatments are unsatisfactory due to toxicity, limited efficacy, high cost and difficult administration. Thus, there is an urgent need to develop drugs that are efficacious, safe, and more accessible to patients. Trypanosomatids, including *Leishmania spp.* and *Trypanosoma cruzi*, have an essential requirement for ergosterol and other 24-alkyl sterols, which are absent in mammalian cells. Inhibition of ergosterol biosynthesis is increasingly recognized as a promising target for the development of new chemotherapeutic agents. The aim of this work was to investigate the antiproliferative, physiological and ultrastructural effects against *Leishmania amazonensis* of itraconazole (ITZ) and posaconazole (POSA), two azole antifungal agents that inhibit sterol C14α-demethylase (CYP51). Antiproliferative studies demonstrated potent activity of POSA and ITZ: for promastigotes, the IC_50_ values were 2.74 µM and 0.44 µM for POSA and ITZ, respectively, and for intracellular amastigotes, the corresponding values were 1.63 µM and 0.08 µM, for both stages after 72 h of treatment. Physiological studies revealed that both inhibitors induced a collapse of the mitochondrial membrane potential (ΔΨ*m*), which was consistent with ultrastructural alterations in the mitochondrion. Intense mitochondrial swelling, disorganization and rupture of mitochondrial membranes were observed by transmission electron microscopy. In addition, accumulation of lipid bodies, appearance of autophagosome-like structures and alterations in the kinetoplast were also observed. In conclusion, our results indicate that ITZ and POSA are potent inhibitors of *L. amazonensis* and suggest that these drugs could represent novel therapies for the treatment of leishmaniasis, either alone or in combination with other agents.

## Introduction

The leishmaniases, which are among the most prevalent neglected tropical diseases, are caused by protozoan parasites of the *Leishmania* genus. The disease is endemic in 98 countries worldwide, and more than 2 million new cases occur annually, with high levels of morbidity and mortality [1]. There are three major clinical manifestations of the disease: visceral, mucocutaneous and cutaneous leishmaniases. Other cutaneous manifestations include diffuse cutaneous leishmaniasis, recidivans leishmaniasis and post-kala-azar dermal leishmaniasis [2]. The pathology caused by *Leishmania* depends on several factors, which include the infecting species and the host immune response [3], [4]. More than 90% of cases of visceral leishmaniasis and cutaneous leishmaniases occur in India, Sudan, Bangladesh, Nepal, Brazil, Afghanistan, Saudi Arabia, Algeria, Iran, Iraq and Syria [1]. In Brazil, *Leishmania amazonensis* is one of the species responsible for the cutaneous form of the disease [5] and it is important for the epidemiology of the leishmaniasis in the Amazon region [6]. When the immune system fails to mount an appropriate response against the parasite, *L. amazonensis* can cause clinical manifestations of diffuse cutaneous leishmaniasis [5]. It is a serious public health problem in Brazil, because the lesions cover a large part of the body, sometimes producing mutilated lesions, and is devastating for the patients, because it is incurable using currently available treatments.

Pentavalent antimonial compounds (e.g., sodium stibogluconate and meglumine antimoniate) have been the drugs of choice for the treatment of leishmaniasis for decades worldwide despite their severe side effects [7], [8]. However, they have been recently discontinued in the India. In addition, antimonials are associated with significant failure and relapse rates, especially in immunocompromised hosts [9]–[11]. Pentamidine and amphotericin B are other parenteral alternatives that can cause significant side effects [8], [12]. Miltefosine (Impavido) is the first oral drug available for treatment of visceral leishmaniasis in India [12]–[13], but it is teratogenic, and there are indications that resistance to the drug is appearing in endemic areas [13]. In addition, miltefosine also has significant effects against cutaneous leishmaniasis in human [12] and in murine models of cutaneous leishmaniasis by infection with *L. amazonensis* [14]. Thus, there is an urgent need to develop new drugs that are efficacious, safe, and more accessible to patients.

Sterols are constituents of cellular membranes that are essential for their normal structure and function. Trypanosomatids have an essential requirement for ergosterol and other 24-alkyl sterols, which are absent in mammalian cells [15]–[16], and ergosterol biosynthesis inhibitors (EBIs) have proved to be potential candidates for the treatment of leishmaniasis and other diseases caused by protozoan parasites, such as Chagas disease (17–21). Itraconazole (ITZ) and posaconazole (POSA) are two known azoles that inhibit sterol C14α-demethylase (CYP51), an essential enzyme in the sterol biosynthesis pathway, with potent effects against fungi and trypanosomatids [22]–[28]. In particular, POSA has shown potent activity in murine models of acute and chronic Chagas’ disease [17], [29]. In addition, POSA and ITZ have also been studied in murine models of cutaneous and visceral leishmaniases by infection with *L. amazonensis*, *L. donovani* [18] and *L. infantum* [30]. Furthermore, there are some studies describing the effect of ITZ on patients with cutaneous leishmaniasis [31]–[33]. Thus, we decided to investigate the *in* *vitro* effects of POSA and ITZ on the proliferation and ultrastructure of *L. amazonensis*. We found that both compounds are potent inhibitors of *L. amazonensis* growth and induced multiple severe alterations in the ultrastructure of promastigotes and intracellular amastigotes. In particular, these drugs affected the structure and function of the single giant mitochondrion present in these cells and induced an accumulation of lipid bodies and autophagosomes.

## Materials and Methods

### Ethics Statement

The experiments using animal models to obtain macrophages and *Leishmania* were approved by the Ethics Committee for Animal Experimentation of the Health Sciences Centre, Federal University of Rio de Janeiro (Protocols n. IBCCF 096/097/106), according to the Brazilian federal law (11.794/2008, Decreto n^o^ 6.899/2009). All animals received humane care in compliance with the “Principles of Laboratory Animal Care” formulated by the National Society for Medical Research and the “Guide for the Care and Use of Laboratory Animals” prepared by the National Academy of Sciences, USA.

### Parasites

The MHOM/BR/75/Josefa strain of *L. amazonensis* used in this study was isolated in 1975 by Dr. Cesar A. Cuba-Cuba (Brasilia University, Brazil) from a patient with diffuse cutaneous leishmaniasis and kindly provided by the *Leishmania* Collection of the Instituto Oswaldo Cruz (Code IOCL 0071 - FIOCRUZ). The strain was maintained by inoculation into the base of the tails of Balb/C mice. Axenic promastigotes were cultured at 25°C in Warren’s medium (brain heart infusion plus hemin and folic acid) [34] supplemented with 10% fetal bovine serum (Cultilab, Brazil). Infective metacyclic promastigotes of the Josefa strain were used to obtain intracellular amastigotes in macrophage cultures.

### Drugs and Reagents

Posaconazole (POSA) was provided by the Schering Plough Research Institute (United States). Itraconazole (ITZ) was purchased from Janssen Pharmaceutical Companies (Brazil). Both drugs were dissolved in a 10 mM stock of dimethyl sulfoxide (DMSO) and stored at −20°C. For experiments, new dilutions were prepared in culture medium to ensure that the DMSO concentration in the culture medium did not exceed 0.1%. All the reagents for electron microscopy were from Electron Microscopy Sciences (England).

### *In vitro* Antiproliferative Effects

Promastigote cultures were initiated at a cell density of 1.0×10^6^ cells/ml. After 24 h of growth, POSA or ITZ was added at different concentrations (0.05; 0.1; 0.5; 1; 3; 5; 8 µM for POSA, and 0.05; 0.5; 1; 3; 5; 8 µM for ITZ) from concentrated stock solutions. Cell densities were evaluated daily over 96 h of growth using a Neubauer chamber. To evaluate the effects of POSA and ITZ on *L. amazonensis* intracellular amastigotes, macrophages from the peritoneal cavity of CF1 mice were harvested by washing with Hank’s solution, plated in 24-well tissue culture chamber slides and allowed to adhere to the slides for 24 h at 37°C, 5% CO_2_, in RPMI medium (Gibco, Brazil) supplemented with 10% fetal bovine serum. Adherent macrophages were infected with metacyclic promastigotes at a macrophage-to-parasite ratio of 1∶10 at 35°C, 5% CO_2_, for 2 h and then washed two times with RPMI medium to remove non-ingested parasites. Infected cultures were incubated in RPMI medium supplemented with 10% fetal bovine serum without drugs. After 24 h of infection when the number of amastigotes per macrophage was in the range of two to four, different concentrations of POSA (1; 3; 4; 5; 6 µM) or ITZ (0.01; 0.05; 0.09; 0.5; 1 µM) were added. Fresh medium with POSA or ITZ was added daily for 3 days (72 h of treatment). The cultures were fixed in Bouin’s solution (70% picric acid, 5% acetic acid and 25% formaldehyde in aqueous solution), washed in 70% ethanol and in distilled water. The cultures were then stained with Giemsa for 1 h. To determine the percentage of infected cells, 600 macrophages (infected and non-infected) were counted in a bright field optical microscope using a 100× immersion oil objective. Association indexes (mean number of parasites internalized per cell, multiplied by the number of infected macrophages and divided by the total number of macrophages) were determined and used to calculate the percentage of infection in each study condition. The concentration that inhibited 50% of the growth (IC_50_ value) was calculated and the control infection (without treatment) was used as reference parameter (100% of infection). The results are expressed as the mean of three independent experiments.

### IC_50_ Calculations

For the calculation of the IC_50_ values, percentage of growth inhibition was plotted as a function of drug concentration by fitting the values to a non-linear curve analysis where *f*  =  min + (max − min) / (1 + (x/EC_50_)^Hillslope^), where the IC_50_ is the concentration that inhibits 50% of the growth. The regression analyses were performed with Graphpad Prism 4 Software (United States).

### Cytotoxicity Assay

Cytotoxicity effects of ITZ and POSA against murine macrophages were evaluated using the CellTiter 96® Aqueous MTS Reagent Powder (Promega, United States). Murine macrophages obtained as explained above were cultivated in a 96-well plate with RPMI medium containing 10% fetal bovine serum (Gibco, Brazil) and maintained at 37°C in 5% CO_2_. After 24 h of cultivation, different concentrations of the drugs (5, 15, 25 and 35 µM for POSA and ITZ) were added every 24 h until 72 h of treatment, when the cytotoxicity was measured. Cell viability was assessed by the MTS/PMS assay reaction and results were expressed as optical density measured at 492 nm in a microplate reader and spectrofluorometer SpectraMax M2/M2^e^ (Molecular Devices, United States) [35]. The cytotoxicity concentration to reduce 50% of viable macrophages (CC_50_) was determined.

### Electron Microscopy

Control, ITZ- or POSA-treated promastigotes and intracellular amastigotes inside macrophages were fixed in 2.5% glutaraldehyde in 0.1 M cacodylate buffer (pH 7.2) and postfixed in a solution containing 1% OsO_4_, 1.25% potassium ferrocyanide and 0.1 M cacodylate buffer, pH 7.2. For transmission electron microscopy, cells were dehydrated in acetone and embedded in epoxy resin. Ultrathin sections were stained with uranyl acetate and lead citrate and observed under a Zeiss 900 electron microscope. For scanning electron microscopy, promastigotes were dehydrated in ethanol, critical point-dried in CO_2_, mounted on stubs, sputtered with a thin gold layer and observed under a FEI Quanta 250 scanning electron microscope.

### Estimation of Mitochondrial Transmembrane Electric Potential (ΔΨ*m*)

The mitochondrial membrane potential (ΔΨ*m*) of *L. amazonensis* promastigotes was analyzed after 48 h of treatment with POSA or ITZ (1 and 5 µM), using the JC-1 fluorochrome (Molecular Probes, United States) [36]. This fluorochrome is a lipophilic, cationic, mitochondrial vital dye that accumulates in the mitochondria in response to ΔΨ*m*. At low concentrations, the dye exists as a monomer, which emits at 530 nm (green fluorescence); at higher concentrations the dye accumulates in the mitochondrion and forms J-aggregates, which emit at 590 nm (red fluorescence). Control, POSA and ITZ-treated promastigotes were harvested, washed in PBS, pH 7.2, added to the reaction medium containing 125 mM sucrose, 65 mM KCl, 10 mM HEPES/K^+^ pH 7.2, 2 mM Pi, 1 mM MgCl_2_ and 500 µM EGTA and counted using a Neubauer chamber. To evaluate the ΔΨ*m* for each experimental condition, 1.0×10^7^ parasites were incubated with 10 µg/mL JC-1 for 40 min, with readings made every minute using a microplate reader and spectrofluorometer SpectraMax M2/M2^e^. After 36 min of readings, 2 µM FCCP was added to abolish the ΔΨ*m*. For the positive control, cells were incubated in the presence of 2 µM FCCP, a mitochondrial protonophore. The relative ΔΨ*m* value was obtained by calculating the ratio between the reading at 590 nm and the reading at 530 nm (590∶530 ratio). Each experiment was repeated at least three times in triplicate using a black 96-well plate, and the figures shown are representative of these experiments.

### Evaluation of Membrane Integrity and the Presence of Lipid Bodies via Nile Red Accumulation

Control and treated promastigotes were harvested, washed in PBS, pH 7.2, and counted using a Neubauer chamber. Cells (1.0×10^7^) were then incubated with 10 µg/mL Nile Red (Sigma, Brazil) for 20 min and 1 µM Sytox Blue for 20 min. The experiments were performed in triplicate, using a black 96-well plate. The cells were washed twice before analysis. The final volume in each well was 200 µl of cell suspension in PBS. Readings were taken with a microplate reader and spectrofluorometer SpectraMax M2/M2^e^ using the following wavelengths for excitation and emission, respectively: 485 and 538 nm for Nile Red, and 444 and 560 nm for Sytox Blue. Each experiment was repeated at least three times in triplicate, and the figures shown are representative of these experiments. After the readings, control and treated-parasites incubated with Nile Red were fixed with 4% nascent formaldehyde in 0.1 M phosphate buffer, pH 7.2, before observation under a Zeiss Axioplan epifluorescence microscope using an optical filter set with 450–490 nm for excitation and 528 nm for emission.

### Statistical Analysis

All the graphics were made using the mean of three independent experiments and the bars represent the standard deviation of those. The statistical significance of differences among the groups was assessed using the one-way analysis of variance (ANOVA) test followed by Bonferroni’s multiple comparison test in the GraphPad Prisma 4 Software. Results were considered statistically significant when p<0.01.

## Results

### Susceptibility of *Leishmania amazonensis* to POSA and ITZ

**Figure 1:**
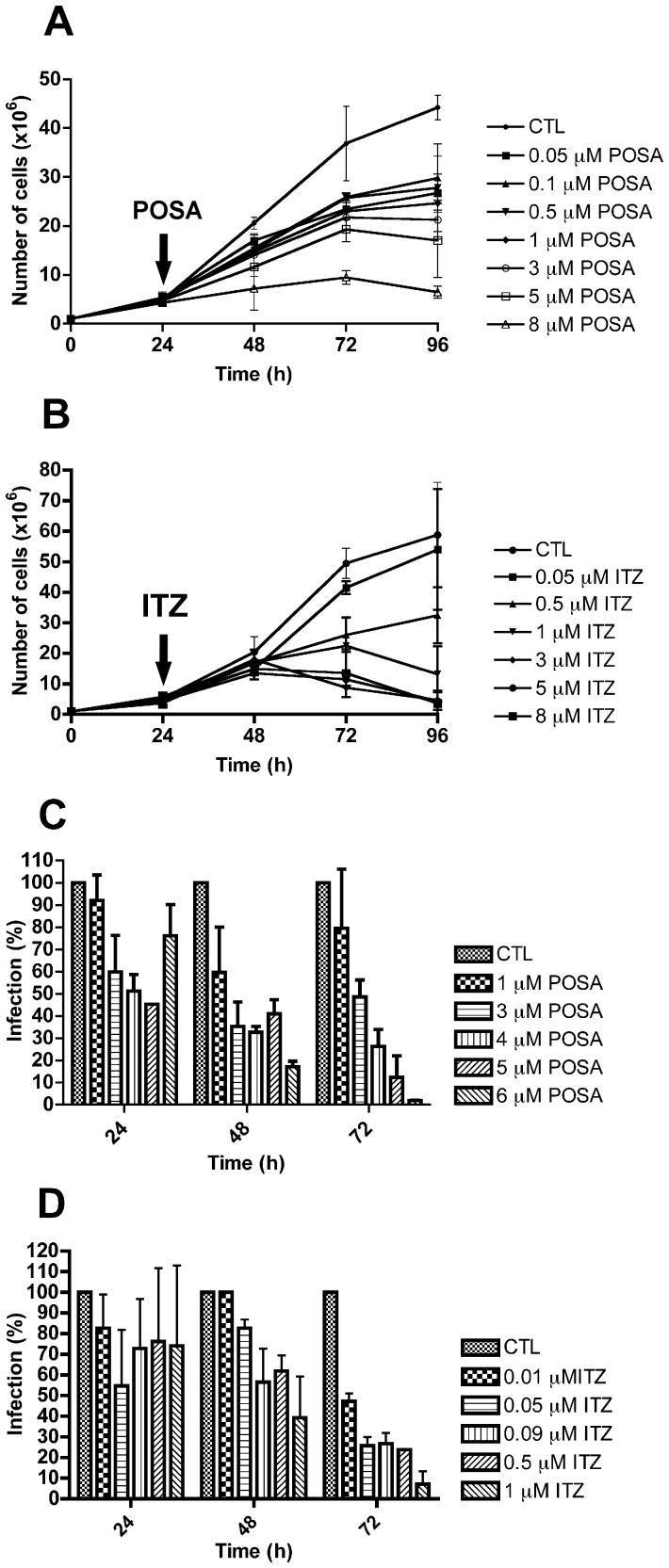
Antiproliferative effects of posaconazole and itraconazole on *Leishmania amazonensis. L. amazonensis* promastigotes (A, B) and intracellular amastigotes (C, D) were treated with posaconazole (POSA) (A, C) or itraconazole (ITZ) (B, D) to evaluate the parasite growth. The arrows indicate the time of the addition of the drugs at the indicated concentrations. The results were plotted as the mean of three independent experiments and the bars represent the standard deviation.

**Figure 2:**
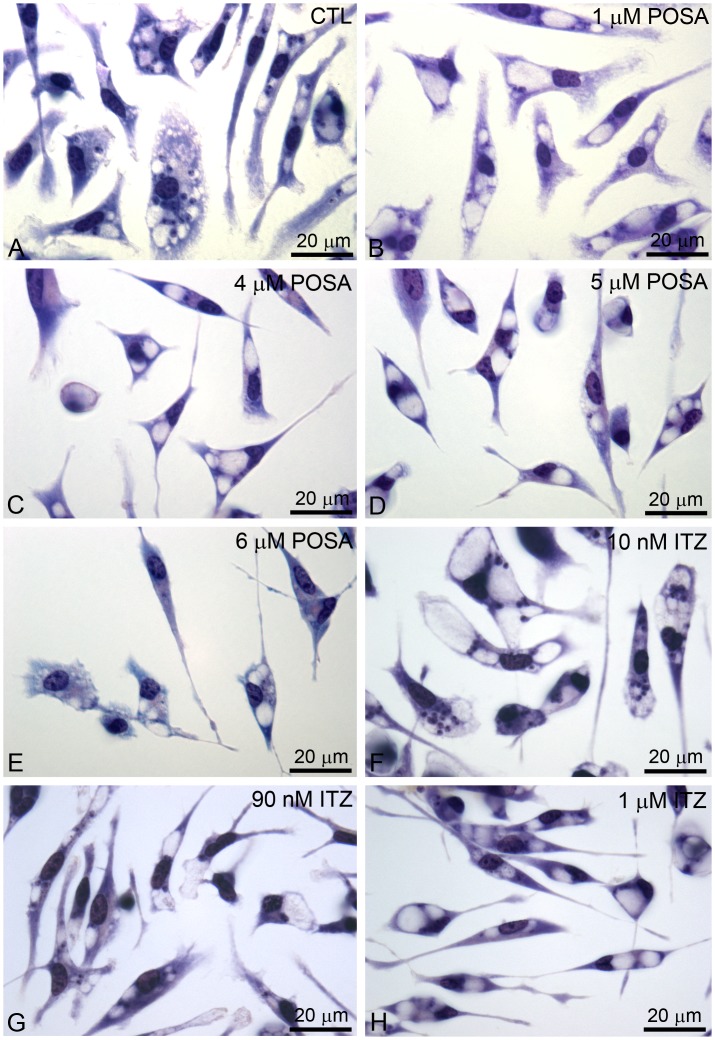
Light microscopy of murine macrophages infected with *L. amazonensis* amastigotes. (A) Control culture with many amastigotes inside parasitophorous vacuoles. (B–H) After 72 h of treatment with different concentrations of POSA and ITZ, a significant reduction in the number of parasites and the presence of several empty parasitophorous vacuoles was observed.

Figure 1 shows the effects of POSA and ITZ on the proliferation of *L. amazonensis* promastigotes and intracellular amastigotes *in vitro*. Both drugs were effective against promastigotes, causing concentration- and time-dependent inhibition of growth. IC_50_ values of 3.94 µM and 0.88 µM were observed for POSA and ITZ, respectively, after 48 h of treatment. After 72 h of treatment, the IC_50_ values were 2.74 µM and 0.44 µM for POSA and ITZ, respectively. When incubated with intracellular amastigotes, the clinically relevant form of the parasite, the effect of the drugs on parasite growth was more potent, with IC_50_ values of 1.95 µM and 0.64 µM for POSA and ITZ after 48 h of treatment, respectively, and 1.63 µM for POSA and 0.08 µM for ITZ after 72 h of treatment. All the IC_50_ values are summarized in table 1. Figure 2 shows several images from bright field optical microscopy of the infections during the treatment. Parasites were not observed after 72 h of treatment with concentrations of POSA >4 µM and concentrations of ITZ >1 µM (Figs. 2C–H). The cytotoxic effects of POSA and ITZ were evaluated against murine macrophages using the MTS assay, and the CC_50_ values are summarized in Table 1. POSA was less toxic than ITZ, with CC_50_ values of 20 µM and 15 µM, respectively. Selectivity indexes were calculated using the IC_50_ values obtained after 72 h of treatment, and ITZ was more selective than POSA *in vitro* (Table 1).

**Table 1:** IC_50_, CC_50_ and selective indexes (SI) for the treatment of *L. amazonensis* promastigotes and intracellular amastigotes with posaconazole (POSA) and itraconazole (ITZ).

	Promastigotes (IC_50_ - µM)		Intracellular amastigotes(IC_50_ - µM)		CC_50_ (µM)*	Selectivity Index (SI)**
Time of incubation	48 h	72 h	48 h	72 h	72 h	72 h
**Posaconazole**	3.94	2.74	1.95	1.63	20	12.2
**Itraconazole**	0.88	0.44	0.64	0.08	15	187.5

* CC_50_ was obtained after 72 h of treatment with both compounds.

** Selectivity Index (SI) was calculated dividing the CC_50_ by the IC_50_ values obtained after treatment for 72 h.

**Figure 3:**
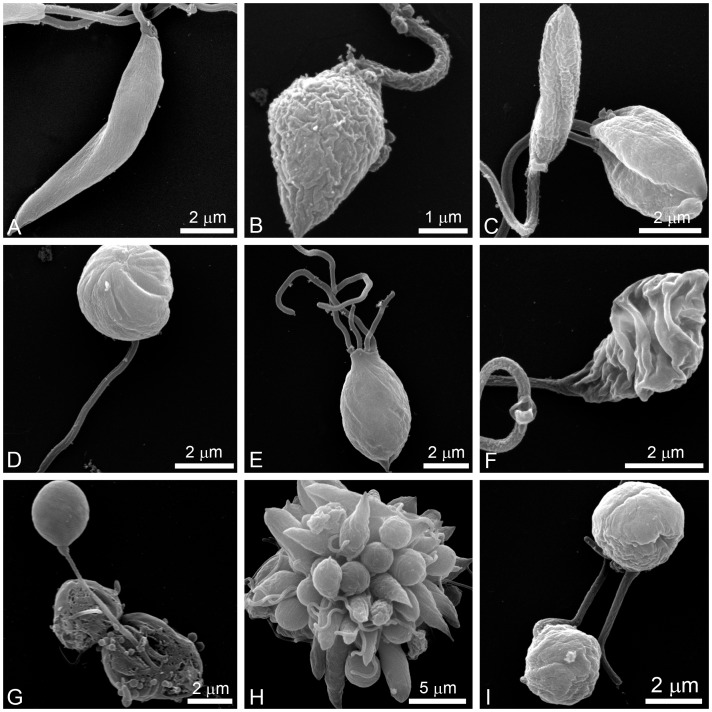
Scanning electron microscopy (SEM) of *L. amazonensis* promastigotes. Control parasites (A) and promastigotes that were treated with different concentrations of POSA and ITZ for 48 h (B–I) were observed by SEM. (B, C) 1 µM ITZ; (D–F) 1 µM POSA; (G) 5 µM ITZ; (H, I) 5 µM POSA. The images show dramatic alterations in promastigote shape (B–I), a promastigote with four flagella (E), and profound changes in the plasma membrane (B, C, F).

Scanning electron microscopy revealed a dramatic alteration in the shape of promastigotes after treatment with POSA or ITZ for just 48 h (Figs. 3B–I). Promastigotes appeared rounded (Figs. 3D, G, H, and I), swollen (Figs. 3B, C, and E) or with cytoplasmic shrinkage (Fig. 3F). In addition, cells with more than one flagellum (Fig. 3E) and changes in the cell surface (Figs. 3B, C, and I) were also observed.

### Effects of ITZ and POSA on Plasma Membrane Integrity and Nile Red Accumulation

**Figure 4:**
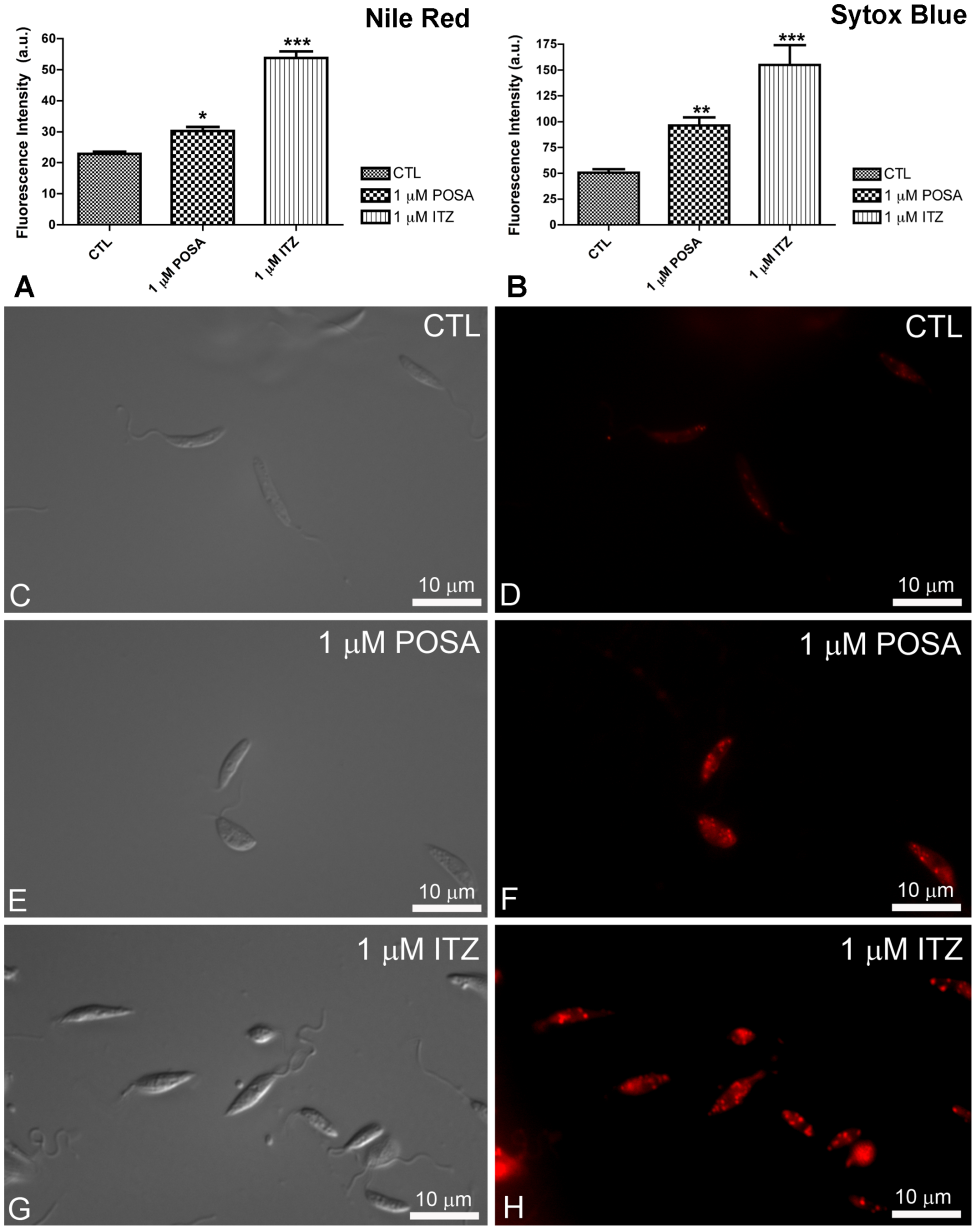
Analysis of lipid body accumulation and plasma membrane integrity in *L. amazonensis* promastigotes. (A–B) Quantitative fluorimetric analysis using Nile Red (A) and Sytox Blue (B). Fluorescence intensity is expressed as arbitrary units (A.U.). The results were plotted as mean of three independent experiments and the bars represent the standard deviation. *p<0.01; **p<0.05; ***p<0.0001. (C–H) Differential interference contrast (DIC) microscopy (C, E, G) and fluorescence microscopy using Nile Red (D, F, H) of control *L. amazonensis* promastigotes and promastigotes treated with 1 µM POSA or ITZ for 48 h. The images demonstrate an accumulation of lipid bodies that are randomly distributed throughout the cytoplasm, confirming the increase in the fluorescence intensity observed in Fig. 4A.

**Figure 5:**
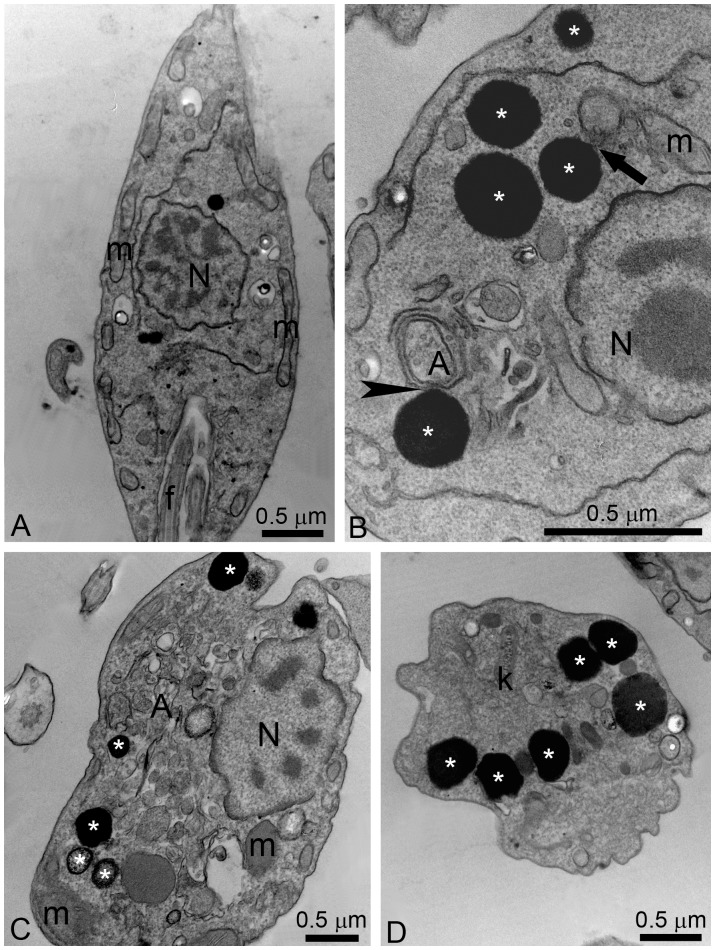
Ultrathin sections of control *L. amazonensis* promastigotes (A) and promastigotes treated with ITZ (B–D). (A) Control promastigotes; (B–C) 5 µM ITZ; (D) 1 µM ITZ. The images show the presence of several electron-dense lipid bodies (asterisks), which sometimes appear near the plasma membrane, the endoplasmic reticulum and mitochondrion profiles (arrow), and autophagosomes (arrowhead). A, autophagosome; f, flagellum; k, kinetoplast; m, mitochondrion; N, nucleus.

To evaluate the effects of EBIs on plasma membrane integrity and Nile Red accumulation, control and treated promastigotes were incubated with Sytox Blue and Nile Red, respectively. Nile Red is a fluorescence marker with special affinity for neutral lipids that become concentrated in the lipid bodies. Sytox Blue is a vital dye with high affinity for nucleic acid that easily penetrates cells with a compromised plasma membrane; thus, it is an efficient dead-cell indicator. Quantitative fluorimetric analysis indicated that treatment with 1 µM POSA and ITZ for 48 h induced significant effects in the accumulation of lipid bodies (Fig. 4A) and in plasma membrane integrity (Fig. 4B). For both analyses, the effects induced by ITZ were greater than those induced by POSA (Figs. 4A, B), congruent with the relative effects of the drugs on promastigote proliferation. Fluorescence images indicated that Nile Red accumulated inside lipid bodies that were randomly distributed throughout the cytoplasm and that the number of lipid bodies markedly increased in drug-treated promastigotes (Figs. 4F, H). Transmission electron microscopy confirmed the presence of lipid bodies after treatment with ITZ and POSA. Lipid bodies were observed in control parasites (Fig. 5A), but in treated promastigotes, several osmiophilic lipid-storage bodies appeared close to the plasma membrane (Fig. 5B–D, asterisks), the endoplasmic reticulum, autophagosomes (Fig. 5B–C, asterisks and arrowhead), and the mitochondrion (Fig. 5B, arrow).

### Effects of POSA and ITZ on Mitochondrial Physiology and Ultrastructure in *L. amazonensis*

The effects of POSA and ITZ on mitochondrial function and ultrastructure were analyzed using two criteria: mitochondrial transmembrane electric potential (ΔΨ*m*), indicated by the JC-1 fluorochrome, and transmission electron microscopy.

**Figure 6:**
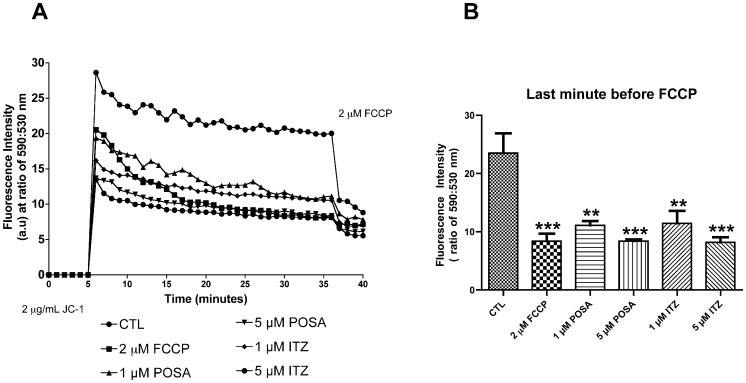
Evaluation of mitochondrial transmembrane electric potential (ΔΨ*_m_*) in *L. amazonensis* promastigotes using the JC-1 fluorochrome. (A) Values of ΔΨ*_m_* were evaluated over 36 min, before the addition of 2 µM FCCP to abolish the mitochondrial potential. Two concentrations of POSA and ITZ were used (1 and 5 µM) for 48 h of treatment. The ΔΨ*_m_* values are expressed as the ratio of the reading at 590 nm (aggregate) to the reading at 530 nm (monomer). (B) Analysis of ΔΨ*_m_* at the last minute before the addition of 2 µM FCCP. The data suggest that similar alterations in ΔΨ*_m_* are induced by POSA, ITZ, and FCCP. The experiments were performed three times, each time in triplicate, and the figures shown are representative of these experiments. **p<0.05; ***p<0.0001.

Promastigotes were treated with 1 and 5 µM POSA and ITZ, respectively, for 48 h prior to the analysis of ΔΨ*m* with JC-1, a cell-permeant cationic, lipophilic fluorochrome. The classic protonophore uncoupler FCCP was used as a positive control to dissipate the mitochondrial electrochemical H^+^ gradient. Simultaneous measurements of J-aggregates (red fluorescence), which accumulate in intact and energized mitochondria, and of J-monomers (green fluorescence) that are a marker for de-energized mitochondria, were used to calculate the ΔΨ*m*, expressed as a ratio of fluorescence intensity obtained at 590 and 530 nm. A decrease in the red:green fluorescence intensity ratio indicates a collapse in the mitochondrial transmembrane potential [36]. Pre-treatment of promastigotes for 48 h with POSA and ITZ at the indicated concentrations led to a marked reduction in the ΔΨ*m* (Figs. 6A, B), which was concentration-dependent (Fig. 6B). To compare the effects of EBIs on the ΔΨ*m* with the effects of a classical inhibitor of mitochondrial metabolism, control (untreated) promastigotes were incubated with 2 µM FCCP during the evaluation of ΔΨ*m* (Fig. 6B), and the observed alteration in the mitochondrial electrochemical H^+^ gradient was similar to that observed in cells pre-treated with 5 µM EBIs.

**Figure 7:**
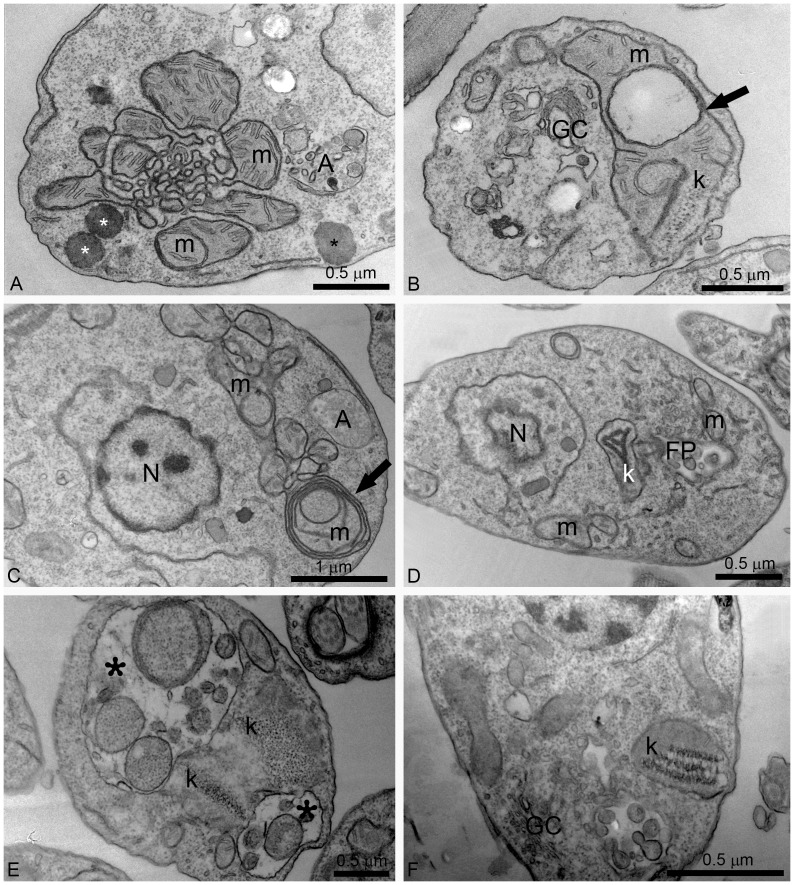
Ultrathin sections of *L. amazonensis* promastigotes treated with different concentrations of ITZ and POSA. (A, B) 1 µM ITZ; (C, D) 1 µM POSA; (E) 3 µM POSA for 48 h; (F) 5 µM POSA for 72 h. Several alterations were observed in the mitochondrion-kinetoplast complex such as: intense disorganization and swelling (A, B, D); alterations in the mitochondrion membranes and the appearance of circular cristae (B, C, arrows); changes in the structure of the kinetoplast (B, D, E, F); and the presence of autophagosomes (A, C, D). In Fig. 7E, two large vacuoles containing membranes and portions of the cytoplasm were observed (asterisks). FP, flagellar pocket; GC, Golgi complex; k: kinetoplast; m, mitochondrion; N: nucleus, A: autophagosome.

Alterations in mitochondrial ultrastructure were also investigated by transmission electron microscopy. Figure 5A shows a control promastigote presenting a normal ramified mitochondrion near the plasma membrane. Treatment with POSA and ITZ induced dramatic alterations of the mitochondrion ultrastructure (Figs. 7A–F). The main alteration observed was a profound mitochondrial swelling, which was followed by remarkable changes in the mitochondrion morphology (Fig. 7A) and its membranes, leading to the appearance of several circular cristae (Figs. 7B–C, arrows). Significant alterations in kinetoplast structure, suggesting a decompaction of the kDNA, were also observed after treatment with both EBIs (Figs. 7B, D–F). In addition, structures similar to autophagosomes (represented by letter A and asterisks in the images) sometimes appeared near the mitochondrion (Figs. 7A, C, E).

### Effects of POSA and ITZ on the General Ultrastructure of *L. amazonensis* Promastigotes and Intracellular Amastigotes

**Figure 8:**
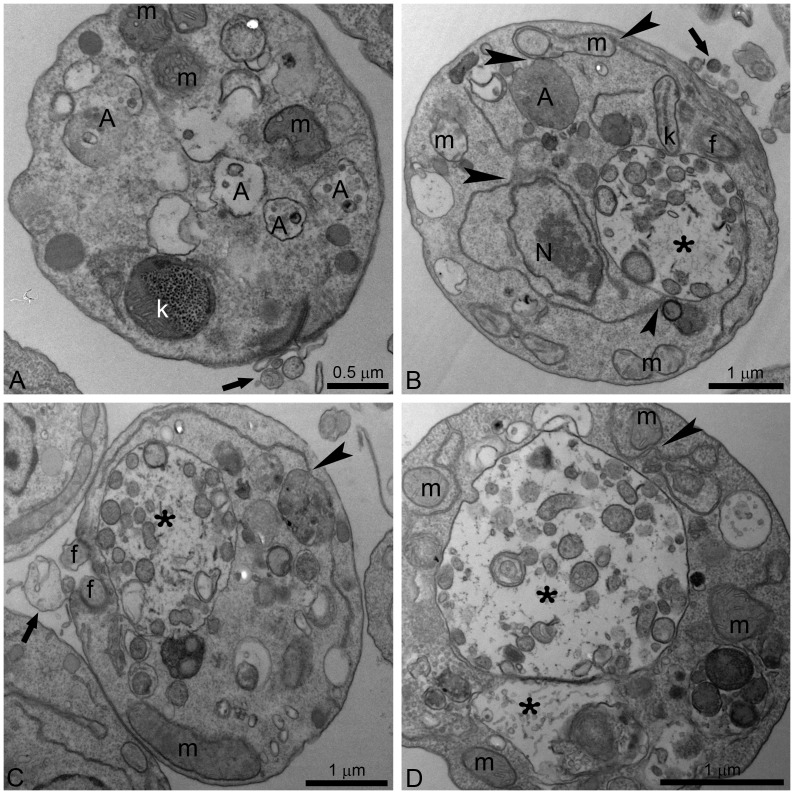
Ultrathin sections of *L. amazonensis* promastigotes. Promastigotes were treated with 1 µM POSA (A, B), and 3 µM POSA (C, D) for 48 h. All images show the presence of small and large vacuoles containing several vesicles, membrane profiles and portions of the cytoplasm (asterisks). The endoplasmic reticulum appears in close association with the nucleus, the mitochondrion and autophagosomes (B–D, arrowheads). In Fig. 8A, changes in kinetoplast structure and vesiculation of the inner mitochondrial membrane were observed. N: nucleus; k: kinetoplast; m: mitochondrion; f: flagellum; A: autophagosome; FP; flagellar pocket.

Alterations in the Golgi complex of promastigotes were observed after treatment with 1 µM ITZ for 48 h (Fig. 7B). In addition, autophagosomes were observed after treatment with different concentrations of POSA for 48 h (Figs. 8A–D). These promastigotes presented a total disorganization of the cytoplasm, with the endoplasmic reticulum appearing in close association with the nucleus, the mitochondrion and autophagosomes (Figs. 8B–D, arrowheads). Several large vacuoles containing many small vesicles and membrane profiles were found in treated promastigotes (Figs. 8B–D, asterisks). In addition, some treated parasites also presented with vesicles leaving the flagellar pocket (Figs. 8A, C, arrows).

**Figure 9:**
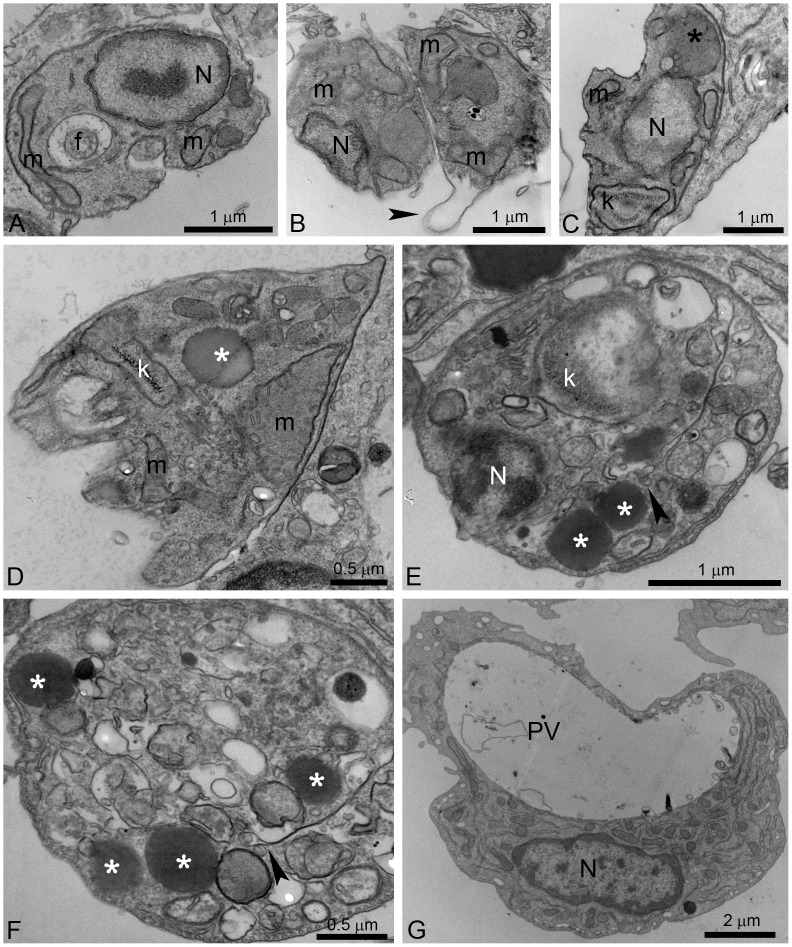
Ultrathin sections of *L. amazonensis* intracellular amastigotes. Control intracellular amastigotes (A) and treated amastigotes with ITZ and POSA (B–G) were observed. (B, C) 500 nM ITZ; (D) 1 µM ITZ; (E–G) 6 µM POSA. Different ultrastructural alterations were observed: mitochondrial swelling (B, D); detachment of the plasma membrane (B, arrowhead); presence of a large megasome (C, black asterisk), lipid bodies (D, E, G, white asterisks) and many vacuoles in the cytoplasm (D, E, G); changes in kinetoplast structure (C, E); and a cell with an empty parasitophorous vacuole (PV) (G). f, flagellum; m, mitochondrion; N: nucleus; PV: parasitophorous vacuole, A: autophagosome, k: kinetoplast.

Ultrastructural alterations were also observed in *L. amazonensis* intracellular amastigotes after treatment with both EBIs (Figs. 9B–G). Figure 9A shows a control amastigote displaying normal ultrastructure of the plasma membrane, the nucleus, the mitochondrion, and the flagellum. Figure 9B shows changes in the plasma membrane that suggest the membrane is detached from the cytoplasm (arrowhead). Different alterations in the mitochondrion were also observed, such as mitochondrial swelling (Fig. 9D) and changes in the kDNA structure (Figs. 9C, E). Megasomes containing membrane profiles (Fig. 9C, asterisk), lipid bodies randomly distributed throughout the cytoplasm (Figs. 9D–F, asterisks) and parasites with abnormal chromatin condensation (Fig. 9E) were also observed. In Figures 9E–F, a close association of lipid bodies with the endoplasmic reticulum (arrowheads) and the mitochondrion can be observed. Finally, macrophages also presented with many empty vacuoles (Fig. 9G).

## Discussion

POSA and ITZ are known azole antifungals that inhibit ergosterol biosynthesis at the level of sterol C14α-demetilase (CYP51). Previous studies demonstrated that POSA has potent antiparasitic activity in a murine model of cutaneous leishmaniasis caused by *Leishmania amazonensis*, although it was less active against visceral leishmaniasis caused by *Leishmania donovani* [18]. In addition, POSA was also effective in the treatment of a patient with cutaneous leishmaniasis caused by *L. infantum* [30], and ITZ was effective in the treatment of cutaneous leishmaniasis [32]–[33]. POSA is currently undergoing phase II clinical trials for the treatment of chronic Chagas disease. Thus, it is important to better understand the effects of these azoles against *L. amazonensis*, which causes infections that respond poorly to standard therapies.

In this study, we confirmed the antiproliferative effects of POSA and ITZ against *L. amazonensis* promastigotes and intracellular amastigotes. Our results are similar to those previously obtained after *in vitro* treatment of *L. amazonensis* promastigotes with POSA [18]. The observed antiproliferative effects indicated that POSA and ITZ were more potent against intracellular amastigotes than promastigotes, and ITZ was more active than POSA against both developmental stages. However, it is important to point out that the experimental condition for each stage is different, which could contribute to the differences in susceptibility observed between them. Against pathogenic fungi, POSA was more efficient than ITZ, with IC_50_ values varying from one to eight times lower than those found for ITZ, depending on the species [37]. The concentrations of POSA and ITZ required to inhibit *L. amazonensis* growth were similar to those required for antifungal activity in previous studies [22]–[26]. Compared with its anti-*Trypanosoma cruzi* activity, POSA was less active against *L. amazonensis*, with IC_50_ values significantly higher than those previously published (14 nM and 0.25 nM for *Trypanosoma cruzi* epimastigotes and amastigotes, respectively) [17].

In the present study, different techniques, such as fluorimetry, fluorescence microscopy and electron microscopy were used to investigate the cellular and subcellular structures and to identify organelles affected by drug treatments. Scanning electron microscopy (SEM) revealed profound alterations in the shape of *L. amazonensis* promastigotes, which became rounded and swollen, as well as changes in the cell surface. In addition, fluorimetric analysis with Nile Red and Sytox Blue revealed an accumulation of lipid bodies and important alterations in plasma membrane integrity, respectively. The presence of many lipid bodies randomly distributed throughout the cytoplasm of promastigotes and intracellular amastigotes was confirmed by transmission electron microscopy. These lipid bodies sometimes appeared in close association with the mitochondrion and the endoplasmic reticulum, which could be related to their biogenesis or to lipid mobilization and utilization, as described for other eukaryotic cells [38]. In addition, lipid bodies also appeared near autophagosomes, suggesting that these organelles could be acting to remove abnormal lipids, likely precursors of sterol biosynthesis, which accumulate in the cytoplasm during treatment with EBIs. Alterations in the plasma membrane of intracellular amastigotes were also observed by transmission electron microscopy. These effects have been described after treatment of *L. amazonensis* and *T. cruzi* with different EBIs [15], [19], [36], [39], [40], including POSA [41], and may be associated with alterations in the lipid composition of treated parasites [42]. SEM also revealed a possible alteration in the cell cycle; some cells presented with more than two flagella, which indicates aberrant cytokinesis. As shown in a previous study [19], other EBIs had the same effect on the cell division, which could be related to alterations in the composition of certain lipids that regulate the cell cycle, or to alterations in cytoskeletal components involved in this cellular process.

We also observed important alterations in the mitochondrion, such as a significant reduction in the mitochondrial membrane potential (ΔΨ*_m_*) after 48 h of treatment at concentrations near the IC_50_. This reduction was very similar to that observed after incubation of parasites with FCCP, a classical protonophore that dissipates the mitochondrial electrochemical H^+^ gradient. Transmission electron microscopy confirmed the mitochondrial alterations after treatment with different concentrations of either ITZ or POSA. The images suggest an intense remodeling of the mitochondrial membranes, which could be related to depletion of the parasite’s endogenous sterols, as demonstrated for other trypanosomatids after treatment with EBIs [43]. These membranes, in contrast with mammalian mitochondrial membranes, contain high levels of endogenous sterols [44]. These alterations are similar to those observed after treatment with amiodarone, an antiarrhythmic drug that also interferes with ergosterol biosynthesis [17], [36], [40], and other EBIs [20]–[21]. In addition, an interesting alteration in kinetoplast structure was also observed (Fig. 7). In trypanosomatids, the kinetoplast appears physically associated with the mitochondrial membrane and the basal body by thin filaments, which form a complex structure known as the tripartite attachment zone (TAC) that is essential for the positioning of the mitochondrial genome and its correct segregation during cell division [45]. This type of alteration in kinetoplast structure has not been previously described during treatment with EBIs and could result from the fragility of the surrounding mitochondrial membranes. Thus, alterations in the mitochondrial membranes could indirectly explain the changes observed in the kinetoplast.

Treatment with POSA and ITZ also induced an intense accumulation of autophagosomes in promastigotes and amastigotes (Fig. 8). These subcellular structures appear to be engulfing parts of the cytoplasm and are located near important organelles, such as the mitochondrion and the endoplasmic reticulum. The autophagosomes were frequently observed as large structures containing many small vesicles and cellular debris, indicating an intense recycling of abnormal membrane structures, organelles and lipid intermediates that accumulated after drug treatment. Autophagy has been described in different protozoan parasites as an important survival mechanism; however, it is also associated with treatment with several classes of compounds [39], [46]. In *T. cruzi*, it was demonstrated that naphthoimidazoles induce an overexpression of the ATG8 genes, which promotes the induction of autophagy in these parasites [47]. Naphthoimidazoles also induced these effects in *Leishmania*; however, treatment with 3-methyladenine, a classic autophagic inhibitor, indicated that autophagy serves as a survival mechanism and that its inhibition causes an increase in apoptotic cell death in the early hours of treatment [48]. The remodeling of damaged cellular structures could be related to the presence of several megasomes, which are lysosome-like organelles of intracellular amastigotes.

In summary, our results show that ITZ and POSA have a strong antiproliferative effect on *L. amazonensis* promastigotes and intracellular amastigotes. These drugs alter the general ultrastructure and the mitochondrial physiology of *L. amazonensis* and likely trigger the three known phenotypes of cell death: apoptosis, necrosis, and autophagy. Our observations suggest that ITZ and POSA, either alone or in combination, may be effective in the treatment of leishmaniasis.
